# Space Physical Sensor Protection and Control System Based on Neural Network Prediction: Application in Princess Elizabeth Area of Antarctica

**DOI:** 10.3390/s20174662

**Published:** 2020-08-19

**Authors:** Yuchen Wang, Yinke Dou, Jingxue Guo, Dehong Huang

**Affiliations:** 1 College of Electrical and Power Engineering, Taiyuan University of Technology, Taiyuan 030024, China; wangyuchen0204@link.tyut.edu.cn; 2 SOA Key Laboratory for Polar Science, Polar Research Institute of China, Shanghai 200136, China; guojingxue@pric.org.cn (J.G.); huangdehong@pric.org.cn (D.H.)

**Keywords:** unattended, machine learning, long- and short-term memory, lumped parameter method

## Abstract

In the inland areas of Antarctica, the establishment of an unmanned automatic observation support system is an urgent problem and challenge. This article introduces the development and application of an unmanned control system suitable for inland Antarctica. The system is called RIOD (**R**emote Control, **I**mage Acquisition, **O**peration Maintenance, and **D**ocument Management System) for short. At the beginning of this research project, a mathematical model of heat conduction in the surface observation chamber was established, and the control strategy was determined through mathematical relationships and field experiments. Based on the analysis of local meteorological data, various neural network models are compared, and the training model with the smallest error is used to predict the future ambient temperature. Moreover, the future temperature is substituted into the mathematical model of thermal conductivity to obtain the input value of the next input power, to formulate the operation strategy for the system. This method maintains the regular operation of the sensor while reducing energy consumption. The RIOD system has been deployed in the Tai-Shan camp in China’s Antarctic inland inspection route. The application results 4.5 months after deployment show that the RIOD system can maintain stable operation at lower temperatures. This technology solves the demand for unmanned high-altitude physical observation or astronomical observation stations in inland areas.

## 1. Introduction

The purpose of this research project is to combine the heat transfer modelling analysis and the neural network prediction model algorithm to provide environmental prediction and support strategies to support the work of automated observation equipment related to space physics in the region. So far, China has established five research camps in Antarctica, of which the Tai-Shan field in the Princess Elizabeth area is the only inland summer camp. Since Tai-Shan Camp is located inland in Antarctica, 520 kilometres from the coastline, the energy demand and fuel transportation costs are higher than other coastal checkpoints. In the research process of the Antarctic continent, including glaciology, geophysics, astronomy, and other disciplines, an essential reason for its progress is the improvement of data quality. The need for more and better measurements along with advances in technical capabilities is driving the ambition to deploy arrays of autonomous geophysical instrument platforms in remote regions. Researchers have an even more urgent need for observations in the Antarctic region. Due to remote areas, lack of infrastructure, and harsh environments, the current measurement is sparse [1]. The activity of the sun has always been very closely related to the change in the earth’s natural environment. The magnetic interaction between the high-speed solar particle flow (solar wind) and the earth’s magnetosphere is a vital manifestation of the sun’s influence on the ground environment. These interactions can initiate geomagnetic disturbances resulting in geomagnetic storms at Earth that may have an impact on technology and endanger human activity and health [2]. This design serves the physical observation of Earth space. The data obtained during the observation process include aurora, ionosphere, and geomagnetism, which can reflect the activities of the sun and its impact on the earth. There are natural, high-quality observation conditions in the Antarctic inland area, and the development of related physical research in this area is less disturbed by human activities. Taking neutrino detection as an example, the Antarctic is an excellent place for neutrino detection on Earth. The Ice Cube project transforms 1 km^3^ of ultra-transparent Antarctic ice into a particle detector. A total of 5160 optical sensors is embedded into a gigaton of Antarctic ice to detect the Cherenkov light emitted by secondary particles produced when neutrinos interact with nuclei in the ice [3]. However, the premise of this research is to carry out under the circumstances of someone on duty. For some actions that require interannual observations or observations in an unmanned area in the polar night, the unattended remote measurement and control system that replaces people is particularly important. Besides, in the perennial unmanned area, the Antarctic inland environment is more special than other regions, and the unique environment creates excellent observation conditions. Dome A, the summit of the Antarctic Plateau, proved to be an excellent location for optical, near-infrared, and terahertz astronomical observations. Gattini is a wide-field camera installed on the PLATO instrument module as part of the Chinese-led traverse to Dome A in 2009 January [4]. No matter what the system is, we will face the same problem in the interior of Antarctica: the observation in the interior of Antarctica is carried out by automated observation equipment and automated sensors. Moreover, space observations and astronomical observations involve more sophisticated equipment, and the environmental conditions required are different from those of conventional sensors. In the application process, several factors make the deployment of a network particularly complicated; these include difficulties of physical access, extreme climate conditions, potential vandalism, as well as data quality and remote data access in real-time [5]. The system is very stable in the Dome A area. In this research design, Professor Ashley’s excellent field plan was also borrowed. Because of Ashley’s pioneering experiments, we can learn from the advantages of many of its programs. Different from that scheme, the system designed in this paper introduces an unmanned large-scale new energy network in the inland, and the environmental forecast and support strategy of the observation cabin combined with an artificial intelligence algorithm.

The observation plan was specially designed during the research project under the framework of the Antarctic High-Altitude Physics Research Program of the China Polar Research Center and has been applied to high-altitude physical observations at the Tai-Shan Camp (73°51’ S, 76°58’ E) in the Princess Elizabeth area.

The main goal of this paper is to explore the realization of an unmanned platform for safeguarding the sensing observation system in the Antarctic region. The second section introduces the structure of the entire system. The third section analyzes the surface weather of the Princess area Elizabeth and establishes the heat transfer lumped parameter equation to develop the mathematical relationship between the environment and energy. After comparing the five neural network prediction algorithms, the neural network algorithm suitable for the situation is used to train the model pair. Ambient temperature prediction, combined with the above two methods, has initially established a control method to ensure the continuous low energy consumption of the surface observation chamber and the observation quality of the observation system. The fourth part shows the results of the prediction experiment. Moreover, the overall implementation effect and system performance of the entire site during the unmanned observation period is introduced. The conclusion is presented in the last part.

## 2. System Architecture

### 2.1. Overall System Architecture

**Figure 1:**
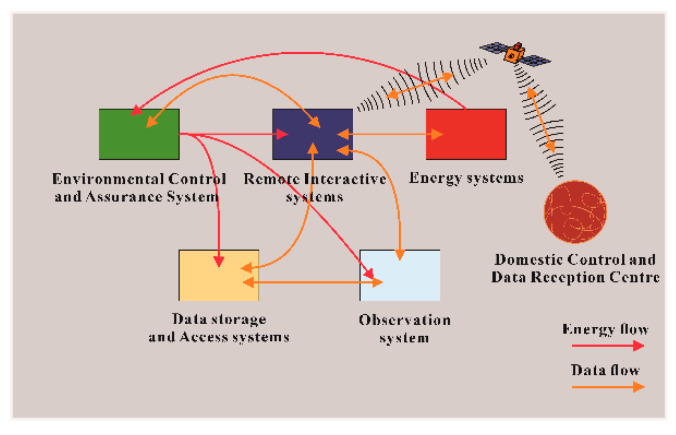
Block diagram of the entire system.

The whole system is composed of five parts: energy control, remote interaction, data storage and access, environmental control, and observation system. The atmospheric parameters, geomagnetism, aurora, and ionosphere in various regions are noted through the observation system. The relevant scientific analysis team will combine this data to analyze the sun’s activity to predict the impact on the earth. The energy control system powers the measurement of the entire observation system, and the remote interactive system provides instructions. The operation status of each observation subsystem and the query and storage of observation data require an environmental control assurance system and a data storage access system to ensure its regular operation. The block diagram of the complete system is shown in Figure 1.

In Figure 1, five parts are marked. Connect any two parts with double arrows or single arrows to indicate the energy and data connection between them. The orange arrow represents the data flow, and the red arrow represents the energy flow. The signal transmission method includes a wired network and wireless network. The transmission method of energy is wired transmission. Currently, it is not necessary to display renewable energy such as wind energy and light energy in the energy transmission system. The direction of the signal flow can be seen. The remote communication and on-site interactive system are interconnected in real-time with the domestic through the satellite network. In addition to the signal connection between the remote interactive system and other systems, there are also interactive signals between the data storage access system and the observation system.

For the energy flow, the energy input of the external environment and the energy loss of the system are omitted in Figure 1. The red energy flow only expresses the transmission of electrical energy between systems. The energy storage part of each system itself is included in the system. The total power generation part of the entire system is included in the energy system, and the distributed power part is included in the environmental protection control system. As the on-site signal gathering place, the environmental protection control system and the other four systems have separate two-way signal transmission, ensuring that the work of each system can be directly controlled through the remote interactive system in China, and the status parameters and database document information of the system can be directly obtained. The power generated by the energy system is first transmitted to the environmental control system, and the power distribution module of the environmental system distributes the power to the remaining three systems again.

### 2.2. Environmental Control, Data Storage Access and Observation System Hardware Configuration

**Figure 2:**
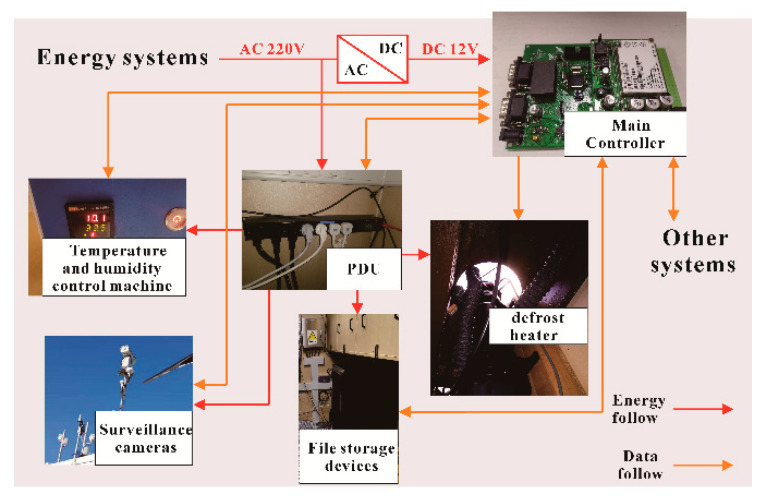
Structure diagram of the environmental protection control system.

The primary function of the environmental control assurance system is to realize the remote image acquisition of the observation room on the ground, the temperature and humidity control in the cabin, the environmental heating, and the power management of the ground equipment. The hardware structure of the system is shown in Figure 2.

The primary sensors include two all-sky imagers: imaging lens observation band 630.0nm (all-sky monochromatic imaging, Keo Scientific Calgary, Calgary, AB, Canada) imager iKon-M (i Kon-M 934 Series, Oxford Instruments, Abingdon, UK) and imaging lens observation band 557.7nm (all-sky monochromatic imaging, Keo Scientific Calgary, Calgary, AB, Canada) imager iKon-M (i Kon-M 934 Series, Oxford Instruments, Abingdon, UK), magnetic through-door magnetometer (LEMI-025, LC ISR, Naukova St., Lviv, UKR), inductive magnetometer (LEMI-120, LC ISR, Naukova St., Lviv, UKR), and TEC ionospheric scintillation Instrument (CJW-1H, CETC Xin Xiang, He Nan, China). The file storage system is a NAS file storage (TS869L, QNAP, Xintai 5th Rd, Xizhi City, Taipei, China), a network switch (TL-SG3226, TP-LINK, Guangming New District, Shenzhen, Guangdong, China) GPS timing instrument (DNTS-8, Neutron, Tong Zhou District, Beijing, China). The related equipment types of the sensing observation system and file storage system are shown in Table 1. 

## 3. Method and Model Preparation

In the Princess Elizabeth area in the east of Antarctica, we obtained the temperature profile data of 20 m under the snow and comprehensive data from surface weather stations. Meteorological data provides a reference and analysis basis for the layout of ground observation facilities and the training of neural network algorithms. According to the theory of heat conduction, a model is established to determine the method of heating power and its relationship to maintaining the internal environment of the interior surface cabin. From the data observed by the weather station in 2016, an interannual outdoor temperature data (hourly temperature data from December 24, 2015, 11:05 to December 30, 2016, 15:58) is selected as the total data set of the neural network model, the sliding time window is set to 20, the ratio of the training sample to the test sample is 0.8 and 0.2, and the number of repeated experiments is five times.

### 3.1. Surface Meteorological Analysis of Taishan Camp in Princess Elizabeth, Antarctica

In the Princess Elizabeth area, Tai-Shan Camp (76°56′54.17″ E, 73°52′21″ S) installed an automatic weather station in 2015. The relevant information of sensors and automatic observation modules carried by the meteorological automatic monitoring station are shown in Table 2 (including the manufacturer, region, communication method, and sensor accuracy).

**Figure 3:**
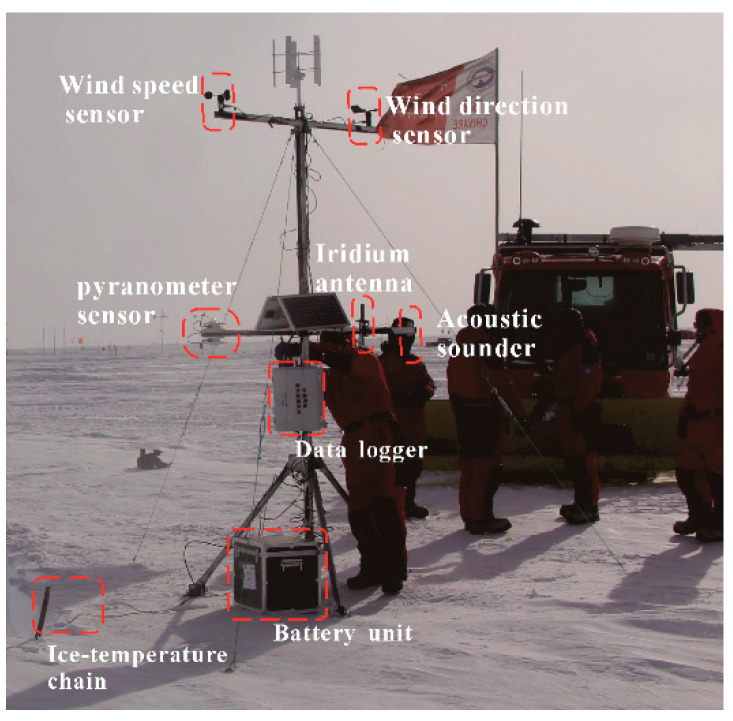
Meteorological observatory deployed in the Antarctic inland during the 2016 Chinese National Antarctic Research Expedition.

The four-blade screw propeller of the wind speed sensor generates the AC sine wave voltage through rotation [6]. The temperature accuracy of the sensing unit EA00 can be detected as 0.5 °C. Forty-one sensing units collect the ice temperature chain sensor at a distance of 0.5 m. The temperature accuracy is sufficient to provide a good temperature gradient for the layout of the underground energy cabin—the installation location of the automatic weather monitoring station (76°58′19.19″ E, 73°51′5.25″ S). The on-site installation situation is shown in Figure 3.

**Figure 4:**
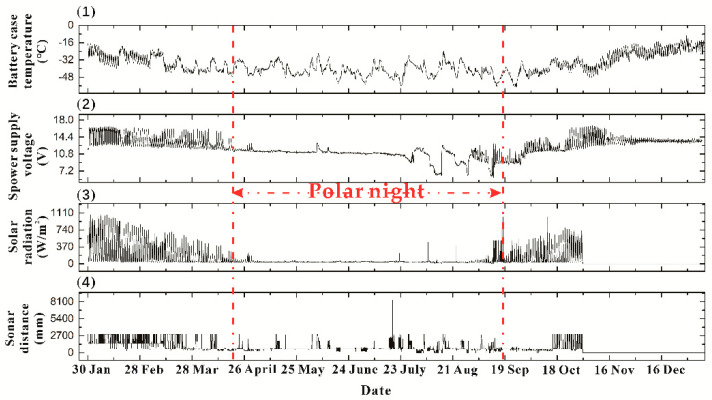
(1) Battery case temperature, (2) Power supply voltage, (3) Solar radiation, and (4) Sonar distance data observed by the weather station installed at Mount Tai in TYUT in 2016.

When installing the temperature profile sensor, the snowmobile PB300 (**P**isten **B**ully–**300** Polar) pushed a 5-m snow pit, and then manually performed hot-melt drilling to obtain a 15-m deep snow hole. The ice temperature chain is placed inside the drill hole. The wind speed and direction sensor is located at the height of 3 m from the snow surface, and the ranging sonar sensor is situated at an altitude of 2 m from the snow surface. Also at this height is the light irradiance sensor, which is installed two at the same height. Our primary concern is the interannual changes of the following two parameters, the temperature of the battery box surface and the voltage of the power supply. It can be seen in Figure 4 that the battery voltage continues to fall during the polar night. Two wind power supplements make the voltage rise back into the perpetual day, and the tension rises to about 14.4 V. During the perpetual day, it can be seen from Figure 4 that the surface temperature of the battery box, the battery voltage, and the range and frequency of light irradiance change are higher than that of the polar night, because the solar radiation intensity changes daily. The point at which each type of data changes from rising to falling represents the most irradiated moment of the day. It can be seen from the data throughout the year that the surface temperature of the battery box has reached –56 °C.

**Figure 5:**
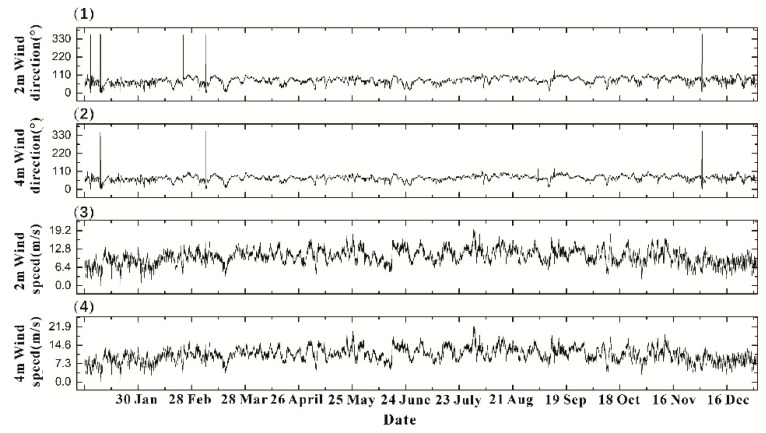
(1) 2m wind direction, (2) 4m wind direction, (3) 2m wind speed, and (4) 4m wind speed. Wind speed and wind direction data of meteorological tower at Tai-Shan station of Chinese Academy of Meteorological Sciences in 2016.

The meteorological station of the Chinese Academy of Meteorological Sciences measures the wind speed and direction 200 m northeast of this weather station. At the same time, the wind speed and direction data are shown in Figure 5. The wind speed and wind direction data of 2m and 4m height were obtained, respectively. It can be seen that the highest wind speed of 19.7 m/s at 2 m appeared on July 30. At this time, the annual maximum wind speed at 4 m above the ground was 22.4 m/s. The minimum wind speed at the two altitudes appeared on January 9 at 0 m/s. The annual average wind speed at 4m height is 10.84 m/s, and the average yearly wind speed at 2 m is 10.17 m/s. It can be seen from Figure 5 that the annual wind direction is mainly distributed between 40° and 100°and combined with the data, the average yearly wind direction at 2 m is 76.94° and 69.61° at 4 m. The average wind speed and direction obtained here provide environmental conditions for the subsequent establishment of the physical model. The physical model defaults the annual wind speed and direction as the average value for calculation.

The wind direction data gives the characteristics of the on-site wind field, based on which the most suitable placement method for the ground observation module is found. Comprehensive weather data predicts that the local factors that affect the regular operation of the equipment may include the following:

1. The low-temperature environment during the polar night makes the voltage of the lead-acid wound battery continue to decrease. The transmitting current is not enough to make the Iridium module transmit. This is the most critical one. We use the acquired temperature information to heat the equipment to avoid this phenomenon.
2. There are strong winds in the Princess Elizabeth area in the east of Antarctica, which has a significant impact on wind speed and direction and ground optical observation equipment.
3. Continuous high radiation intensity may cause ageing of the equipment’s non-metallic enclosure.
4. In the absence of supplementary energy in the photovoltaic system, the wind turbine has a high probability of failure, resulting in no supplement of renewable energy. In the case of the continuous low battery voltage, the battery is eventually damaged and the system cannot be restarted.

### 3.2. Heat Conduction Model and Heating Strategy

The environmental temperature control of the surface observation cabin is the first condition for the ordinary observation of the observation system, but Antarctica is different from the domestic environment, without a real-time weather forecast. Because it is located inland of Antarctica. The frequency of meteorological observations is low, and the accuracy brought by satellite observations may not meet the needs of surface climate observations. Heating control devices that cannot predict the future temperature mainly have the following three problems:

- The first affected object is the dome of the acrylic full-sky imager because this device is different from other observation devices in the South Pole. A cabin temperature higher than 5 degrees Celsius will cause water vapor to rise and condense inside the observation hood, affecting the operation of the all-sky imager.
- The higher the temperature difference will bring more heat loss to the system. During the winter, the fan is self-locking. During the wintering period (that is, from mid-March to mid-August each year), all electrical equipment is only powered by diesel engines. Daily power and diesel demand need to be compressed to a minimum.
- If you set a self-heater with a threshold in the room, for example, if the temperature threshold is set from 0 to –15 °C, the temperature will continue to be –15 °C. The extreme temperature drop or sudden strong wind outside the pole at night will cause the situation. The heat loss in the cabin is too fast. Due to the slow heat transfer of the air commutation, the equipment that is powered on will be shut down and cannot be restarted because the temperature is too low. Real-time classic control algorithms, such as PID, are more cumbersome in the parameter adjustment process. On the one hand, the heat transfer in the air is difficult to calculate in addition to radiant heat; on the other hand, the time of convective heat transfer is not easy to determine.

Therefore, the on-site heating strategy is studied by establishing a lumped parameter model of the surface observation cabin, and the relationship between the temperature difference and the heating power is determined by combining the relationship between the model parameters and the experimental field data.

#### Construction of Lumped Parameter Model and Establishment of Mathematical Relationship

**Figure 6:**
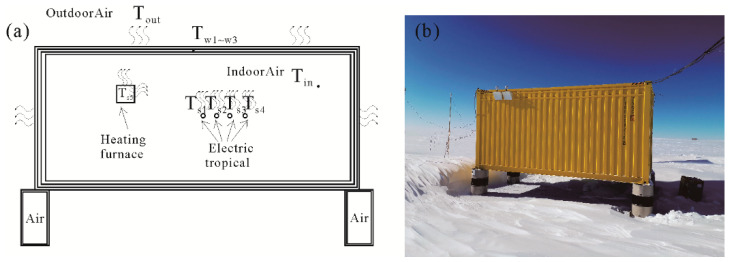
(**a**) The distribution of thermal nodes in the surface observation chamber. (**b**) The actual arrangement of the surface observation chamber at the Antarctic site in December 2019.

First, a lumped parameter model that conforms to the surface observation module is built. The heating model diagram and on-site photos are shown in Figure 6. 

The temperature of observation cabin is affected by many factors, and the reflection of the snow surface and the solar radiation is discarded (to ensure the lower temperature limit of the system). Because the cabin is closed, the internal equalizing fan can ensure the uniform distribution of the indoor air temperature in the space. Figure 6a is a diagram of the thermal network node of the research object, and Figure 6b is the actual diagram of the research object.

**Figure 7:**
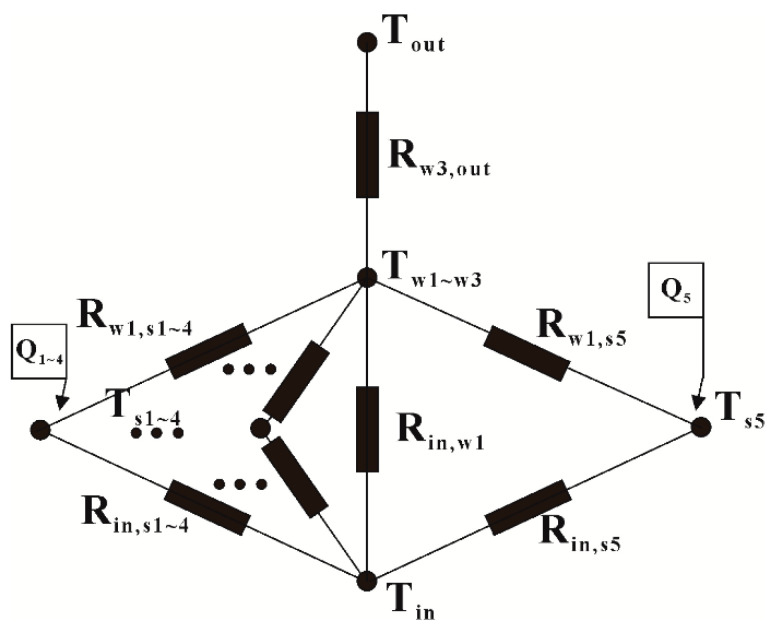
Heat transfer lumped parameter equation of surface observation cabin.

Since l/λ≪1/h, l is half the thickness of the observation cabin wall, λ is the thermal conductivity and h is the surface heat transfer coefficient. The entity satisfies the lumped parameter method and can construct a lumped parameter temperature node model of the surface observation cabin. The equalizing fan and the normal heating placement position make each lumped internal temperature difference extremely small, simplifying a thermal system into a few discrete “lumps”. Among them, T_s1_-T_s5_ respectively represent the temperature of heating zone No. 1, No. 2, No. 3, No. 4, and No. 5 of the electric heating furnace. $T_{w1},T_{w2},T_{w3}$ represent the temperature of the entire inner wall, interlayer, and outer wall, respectively. Moreover, $T_{outdoor}$, $T_{in}$ refer to the temperature of outside air and inside air. As such, we can obtain the thermal network map as shown in Figure 7.

The nodal temperature equation of air $T_{in}$ is:

(1) $$\left(m_{in}c_{in}\right)\frac{dT_{in}}{dt}=\frac{T_{w1}-T_{in}}{R_{in,w1}}+Q_{in},$$

where $T_{in}$ = internal air node temperature; $T_{w1}$ = internal wall node temperature; $m_{in}$ = total inner air mass; $c_{in}$ = civil air specific heat capacity; and $R_{in,w1}$ = thermal resistance between interior air and interior wall. The second term on the right side of Equation (1) represents the total heat input from the surface observation chamber.

Similarly, the nodal equations for the inner wall, interlayer and outer wall can be obtained. The control equation of the interior wall node is:

(2) $$\begin{array}{ll} \left(m_{w1}c_{w1}\right)\frac{dT_{w1}}{d\tau } & =\frac{T_{in}-T_{w1}}{R_{in,w1}}+\frac{T_{w2}-T_{w1}}{R_{w1,w2}}+\frac{E_{s1}-E_{w1}}{R_{s1,w1}}\\ & +\frac{E_{s2}-E_{w1}}{R_{s2,w1}}+\frac{E_{s3}-E_{w1}}{R_{s3,w1}}\\ & +\frac{E_{s4}-E_{w1}}{R_{s4,w2}}+\frac{E_{s5}-E_{w1}}{R_{s5,w2}} \end{array}.$$

The control equation of mezzanine node is:

(3) $$\left(m_{w2}c_{w2}\right)\frac{dT_{w2}}{d\tau }=\frac{T_{w1}-T_{w2}}{R_{w1,w2}}+\frac{T_{w3}-T_{w2}}{R_{w2,w3}}.$$

The control equation of external wall node is:

(4) $$\left(m_{w3}c_{w3}\right)\frac{dT_{w3}}{d\tau }=\frac{T_{w2}-T_{w3}}{R_{w2,w3}}+\frac{T_{out}-T_{w3}}{R_{w3,out}}.$$

$E_{x}$ in the above control equation is the emission power of a black body at temperature $T_{x}$. There are following equations to solve the specific energy. The meaning of this expression is radiation heat flux between the surface of an object with temperature $T_{a}$ and object with temperature $T_{b}$. Where σ = blackbody radiation constant; ε = emissivity; A = surface area; $X_{a,b}$ = radiation angle coefficient from surface a to b; $E_{a,b}$ = emission power of blackbody at temperature T; and $R_{a,b}$ = radiation heat transfer resistance between surfaces a and b.

(5) $$q_{f}=\frac{\sigma T_{a}^{4}-\sigma T_{b}^{4}}{\frac{1-\varepsilon_{a}}{\varepsilon_{a}A_{a}}+\frac{1}{A_{a}X_{a,b}}+\frac{1-\varepsilon_{b}}{\varepsilon_{b}A_{b}}}=\frac{E_{a}-E_{b}}{R_{a,b}}.$$

From Equations (1)–(5), we can see that the surface area, radiation angle, and other parameters do not change with the temperature. The emissivity and temperature of the material in a small range of low-temperature changes linearly and the proportional coefficient can be regarded as a constant.

Before pushing to the following formula, we must introduce a concept, called excess temperature, which refers to the temperature difference between two adjacent nodes at time t. It can be expressed as θ, for example, the excess temperature of $T_{in}$ and $T_{w1}$ at time 0 is $\theta_{0}=T_{in}{}_{\left(0\right)}-T_{w1}{}_{\left(0\right)}$. From this, we can obtain the following equation:

(6) $$m_{in}c_{in}\frac{d\theta }{dt}=-\frac{\theta_{0}}{R_{in,w1}}+Q_{in}=-\frac{\left(T_{in}{}_{\left(0\right)}-T_{w1}{}_{\left(0\right)}\right)}{\frac{1-\varepsilon_{in}}{\varepsilon_{in}A_{in}}+\frac{1}{A_{in}X_{in,w1}}+\frac{1-\varepsilon_{w1}}{\varepsilon_{w1}A_{w1}}}+Q_{in}.$$

Thus, the equation can be obtained from Equation (6):

(7) $$\overset{\theta }{\underset{\theta_{0}}{\int }}\frac{d\theta }{\theta }=-\overset{t}{\underset{0}{\int }}\frac{1-Q_{in}R_{in,w1}/\theta }{c_{in}m_{in}R_{in,w1}}dt.$$

Thus, the equation to be iterated for the first time is obtained:

(8) $$\frac{\theta }{\theta_{0}}=\frac{T_{in\left(t\right)}-T_{w1(t)}}{T_{in}{}_{\left(0\right)}-T_{w1\left(0\right)}}=\exp\left(-\frac{1-Q_{in}R_{in,w1}/\theta }{m_{in}c_{in}R_{in,w1}}t\right).$$

Through Equation (8), the mathematical relationship between the time, the temperature difference between the indoor air and the inner surface, and the heat input are established. Since the outdoor temperature is known (because we have adopted a neural network to predict the temperature of the object for 24 h, after doing so, we assume that the average temperature in the next hour is known and unchanged), we can establish the equation:

(9) $$\left(T_{in\left(0\right)}-T_{outdoor\left(0\right)}\right)-\left(T_{in\left(t\right)}-T_{outdoor\left(t\right)}\right)=T_{in\left(0\right)}-T_{in\left(t\right)}=\left(\theta_{0}-\theta \right).$$

By the heat equation, we can get the indoor temperature $T_{in}$, the time t, and the heat input $Q_{in}$ relationship. The comparison is as follows:

(10) $$\begin{array}{ll} Q_{in}-Q_{out} & =mc\left(T_{in\left(0\right)}-T_{in\left(t\right)}\right)=mc\left(\theta_{0}-\theta \right)\\ & =mc\theta_{0}\left(1-\frac{\theta }{\theta_{0}}\right)=mc\theta_{0}\left[1-\exp\left(-\frac{1-Q_{in}R_{in,w1}/\theta }{m_{in}c_{in}R_{in,w1}}t\right)\right]\\ & =mc\theta_{0}\left[1-\exp\left(-\frac{1}{m_{in}c_{in}R_{in,w1}}t+Q_{in}t/\theta \right)\right]\\ & =0 \end{array}.$$

In the field test heating experiment, a set of heating data with constant temperature difference under no wind condition is selected (the data sampling frequency is 15 min/time indoors and 1 h/times outdoor). At this time, the heating power is 383 W, reaching thermal equilibrium $\left(\theta =\theta_{0}\right)$. After two iterations, thermal stability is reached and Equation (11) can be determined. At this time, the excess temperature is set as $\theta =T_{in\left(t\right)}-T_{outdoor\left(t\right)}$, and Equation (11) is written as:

(11) $$\begin{array}{ll} Q_{in}-Q_{out} & =mc\left(T_{in\left(0\right)}-T_{in\left(t\right)}\right)=mc\left(\theta_{0}-\theta \right)\\ & =mc\theta_{0}\left(1-\frac{\theta }{\theta_{0}}\right)=mc\theta_{0}\left[1-\exp\left(-\frac{1-Q_{in}R_{in,outdoor}/\theta }{mcR_{in,outdoor}}t\right)\right]\\ & =mc\theta_{0}\left[1-\exp\left(-\frac{1}{mcR_{in,outdoor}}t+Q_{in}t/\theta \right)\right]\\ & =0 \end{array},$$

where $Q_{in}$ and $Q_{out}$ represent the input and output energy of the entire system,$R_{in,outdoor}$ represents the equivalent thermal resistance between internal air and external air, and $m_{s}c_{s}$ represents the product of the mass and specific heat of the equivalent thermal resistance itself. Thus, the following is available:

(12) $$Q_{in}/\theta =\frac{1}{m_{s}c_{s}R_{in,outdoor}}.$$

Through the field test data, it can be found that under the heating power of 383 W, the thermal balance is reached when the indoor and outdoor temperature difference is 20 °C. It can be seen from Equation (10) that the heat exchange per unit time is determined by the temperature difference between the outside environment and the initial time. When the heating time is fixed, and the default material properties do not change with temperature, the heating power is linearly related to the temperature difference at the first moment. Based on this, it can be determined that the heating power at different temperature differences has the following table. At this time, the heating method is 1 h; the temperature of the air in the cabin is maintained at around –5 °C. This table applies to other temperature differences. The heating power in Table 3 corresponds to the heating power to maintain the ambient temperature.

The characteristics of air and related materials are shown in Table 4 (the conditions listed are 2600 altitudes, −23 °C). It can be seen that the specific heat of materials Cyanuramide, 2,4,6-Triamino-s-Triazine, Asbestine and PU is the same, because Cyanuramide, 2,4,6-Triamino-s-Triazine in the list is the liquid density under normal pressure, which is a mixture. The thickness is uncertain, so no parameter substitution calculation is performed. It is feasible to determine the heating strategy according to the heat transfer Equation (12).

### 3.3. Experiment Preparation and Machine Learning Model

Next, we need to obtain a prediction of the future temperature, that is, the temperature value within one or several hours in the future. To a certain extent, machine learning models, leveraging the correlations among parameters, can be trained to quickly and automatically identify alterations and faults [7]. The original input time series data formed by time and temperature and the single output time-series data generated by machine learning and prediction have become the main parameters of the future environment in this design. Although many methods use long-term and short-term memory, we use three latest methods of LSTM. We also analyzed the prediction results of the BP neural network and the ELM neural network.

#### 3.3.1. BPNN (Back Propagation Neural Network) & ELM (Long Short-Term Memory)

BP neural network is the most basic neural network, and its output results adopt forward propagation, and errors adopt backward propagation (Back Propagation). BP neural network has a three-layer structure of the input layer, hidden layer, and output layer. The input layer receives data, and the output layer outputs data. The neurons of the previous layer are connected to the neurons of the next layer, information transmitted by the neurons of the previous layer is collected, and the value is passed to the next layer after “activation”.

The weights of the hidden layer nodes of the ELM network are randomly generated or manually defined. The learning process only needs to calculate the output weights. Through such rules, the generalization performance of the model is excellent, and the speed is much improved. The most prominent feature of ELM is that for traditional neural networks, it is faster than conventional learning algorithms under the premise of ensuring learning accuracy.

#### 3.3.2. LSTM (Long Short-Term Memory)

**Figure 8:**
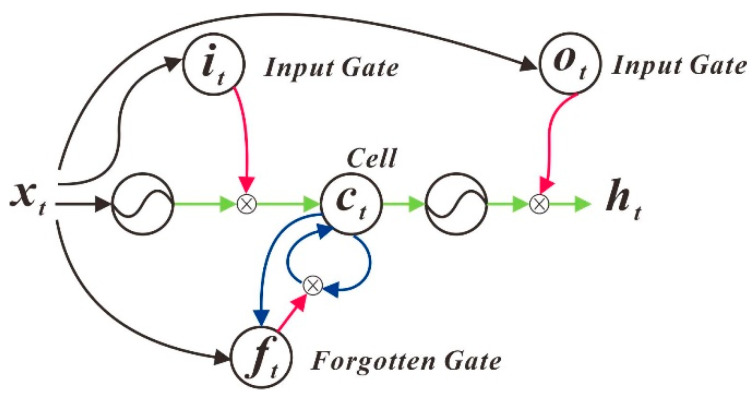
Long Short-Term Memory (LSTM) structure diagram.

The full name of LSTM is Long Short-Term Memory, which is a kind of RNN (Recurrent Neural Network). LSTM is very suitable for modelling time series data due to its design characteristics. Recently, a new neural network was proposed, called LSTM [8]. The three core arithmetic structures contained in LSTM determine that it can achieve long- and short-term memory based on RNN. The algorithm structure is shown in Figure 8.

#### 3.3.3. Bi LSTM (Bi-directional Long Short-Term Memory)

LSTM can only predict the output at the next time based on the timing information of the previous time, but in some problems, the production at the current time is not only related to the former state but may also be associated with the future state. The forward LSTM can obtain the past data information of the input sequence, and the backward LSTM can collect the following data information of the input sequence. [9] This can be expressed by the following formula:

(13) $$\left\{ \begin{array}{l} \vec{h_{t}}=\vec{LSTM}\left(h_{t-1},x_{t},c_{t-1}\right),t\in [1,T]\\ \overset{\leftarrow }{h_{t}}=\overset{\leftarrow }{LSTM}\left(h_{t+1},x_{t},c_{t+1}\right),t\in [T,1]\\ H_{t}=\left[\vec{h_{t}},\overset{\leftarrow }{h_{t}}\right] \end{array}\right..$$

**Figure 9:**
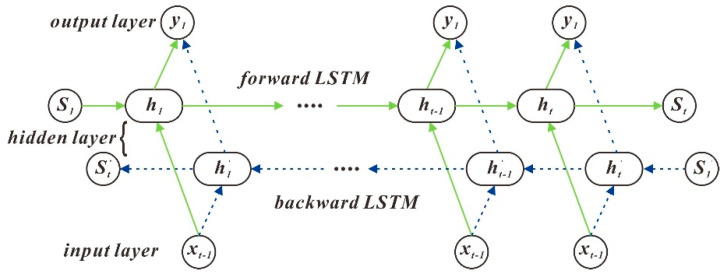
Bi LSTM structure diagram.

The algorithm structure is shown in Figure 9.

#### 3.3.4. SAE (Stacked Autoencoder)

**Figure 10:**
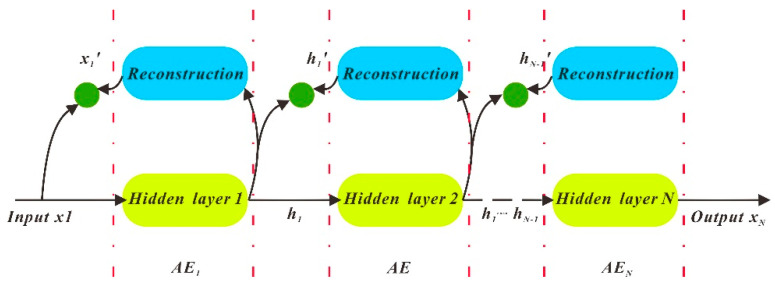
Stacked Autoencoder (SAE) structure diagram.

AE (Autoencoder) has only one hidden layer. More specifically, the input layer and output layer of AE are equal [10]. SAE is the superposition of multiple AEs. After the first AE is executed, subsequent AEs are performed in order until the Nth, and the output result is the SAE superimposition result. The algorithm flow chart is shown in Figure 10:

Table 5 shows the computer configuration.

In the design process of related algorithms, the structure has been introduced in this section. ME, GT, HL1, HL2, AF, ILR, LRDP, LRDF, V, ETF, DTF, L2R, L2WR, SP, MINON, MAXON, MINLN, and MAXLN are the abbreviation of Max Epochs, Gradient Threshold, Hidden Layer 1, Hidden Layer 2, Activation Function, Initial Learn Rate, Learn Rate Drop Period, Learn Rate Drop Factor, Verbose, Encoder Transfer Function, Decoder Transfer-Function, L2Regularization, L2WeightRegularization, and Sparsity Proportion, etc. [8,11,12,13,14] and need to be set. Table 6 shows the settings of the related parameters.

So far, we have completed the model establishment, environment construction, and parameter setting in the prediction experiment. The next step is to prepare the experimental data. First, a data set is selected with a large sample size as a one-dimensional matrix input, and then the values in the matrix are normalized (matrix row minimum and maximum values to [–1, 1]). The set is divided after normalization; 80% of the data (including the data of the validation set) is the training set input, and 20% of the data is the test set input. Subsequent experiments do not require a verification set because the parameters of the test results do not need to be adjusted. In the selection process of the settings in Table 6, we refer to the research methods of long-term memory and bio-signal feature compression to identify negative emotions. In the comparison of algorithms [15], the next section analyzes related training results and experimental results.

## 4. Results

### 4.1. Optimal Model Result

**Figure 11:**
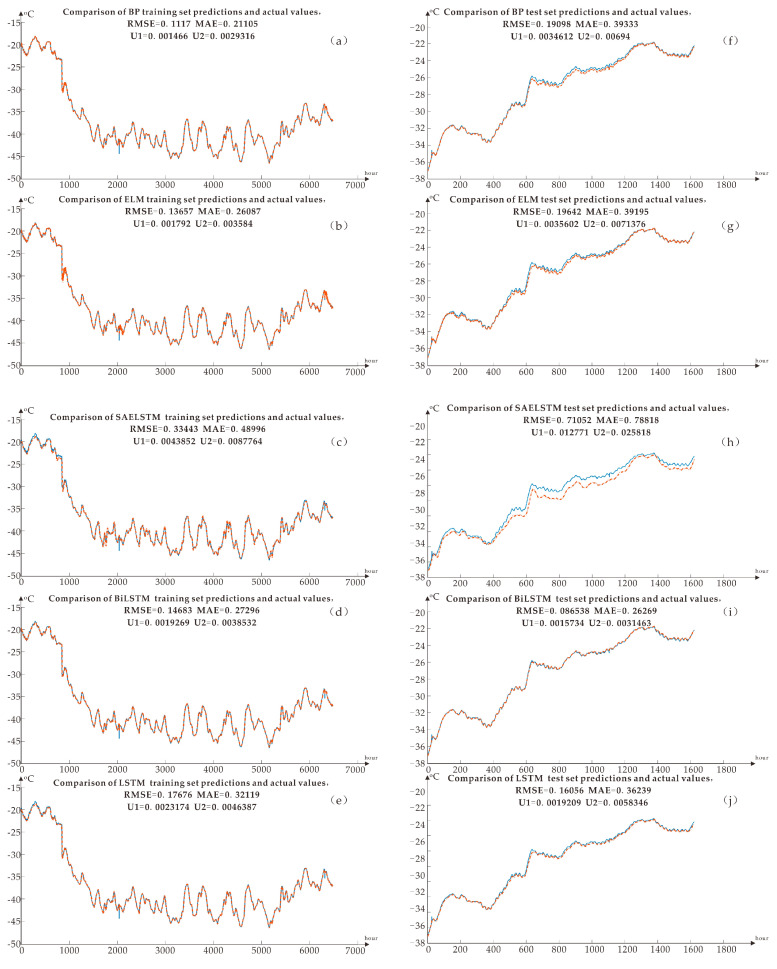
(**a**) BP neural network training set training result (**b**) ELM neural network training set training result (**c**) SAELSTM neural network training set training result (**d**) Bi LSTM neural network training set training result (e) LSTM neural network training set training Results (**f**) BP neural network test set training results (**g**) ELM neural network test set training results (**h**) SAELSTM neural network test set training results (**i**) Bi LSTM neural network test set training results (**j**) LSTM neural network test set training results.

As shown in Figure 11, the training set and test set of five kinds of neural networks are shown.

There are 8106 sets of data in the data sample; that is, the period is 8106 h, from 11:05 on December 24, 2012, to 7:45 on December 27, 2013. After much training and comparisons, the Bi LSTM error (RMSE, MAE, U1, U2) is the smallest. It is preliminarily believed that the Bi LSTM algorithm model is suitable for training and predicting temperature and weather factors in this area of Antarctica. The performance parameters of each model shown in Figure 11, including RMSE (Root Mean Square Error) and MAE (Mean Absolute Error) which are shown in each small graph. Compared with other algorithms, the RMSE of the Bi LSTM training set is 0.14683, which is the minimum of all root mean square errors. Because the training set RMSE is too small may be caused by overfitting, but the RMSE of the test set is equal to 0.086538 less than the training set, so the possibility of overfitting is ruled out. We choose Bi LSTM as the method to predict temperature data in the next few hours. However, if a small sample is subsequently used for short-term prediction, the RMSE of other algorithm models is lower. It is recommended to combine Bi LSTM with this minimum prediction algorithm to generate the final operation [16,17,18,19,20].

**Figure 12:**
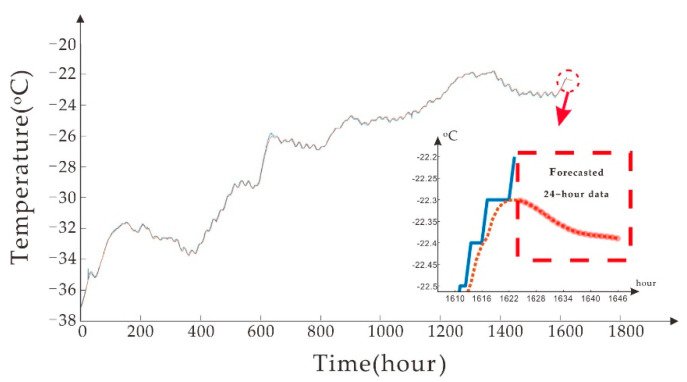
Bi LSTM model predicts the results of the next 24 h of data.

The prediction result is shown in Figure 12, which uses the Bi LSTM training model, and the enlarged part in the chart is the prediction part. In Figure 12, the temperature data for the next 24 h is obtained (blue is sample data, and red is predicted data). Historical data can predict future time series. After comparing different data sizes of different samples, the more complete the time series data, the more accurate the forecast. The primary purpose of Figure 12 is to show the predictive function of the model and the expression of the results.

### 4.2. Operation Ticket Generation

**Figure 13:**
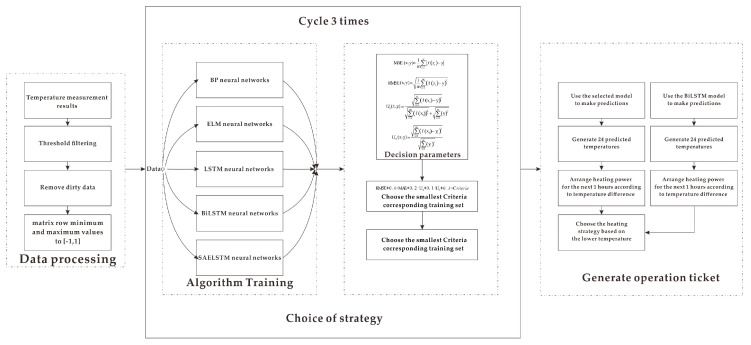
Predictions of 24 data after the training set.

In the process of obtaining temperature data, sometimes the sensor is encountered by the wind and snow. We found that there were a lot of incomplete, inconsistent, and abnormal values in the original temperature profile data from the ice-tethered, seriously affecting the efficiency of the algorithm. During data mining, the original data preprocessing process was also viewed as data cleaning [21]. Figure 13 shows the process of obtaining a heating operation ticket in the cabin. It is roughly divided into three parts:

1. Input sample data: data acquisition, then data preprocessing, unusable data culling, and filtering after thresholding.
2. Determination of the prediction method: Separate the amount of data by 80% and 20%. After normalization and regularization, it is input as a training set and test set. During the training and testing of five neural networks (BPNN, ELM, Bi LSTM, SAELSTM, and LSTM), no overfitting occurred in each system. Loop three times and select the algorithm corresponding to the smallest error.
3. According to the corresponding temperature prediction, based on the current temperature and the temperature in the next two hours, a heating operation ticket is generated, and the lower temperature is used as the predicted temperature to represent the average temperature in the next hour. The prediction result is compared with the prediction result of the Bi LSTM model, and then the result with a lower temperature in the next hour is selected as the parameter generated by the temperature difference. Then check the table and heat accordingly.

### 4.3. RIOD’s On-Site Operation Status

**Figure 14:**
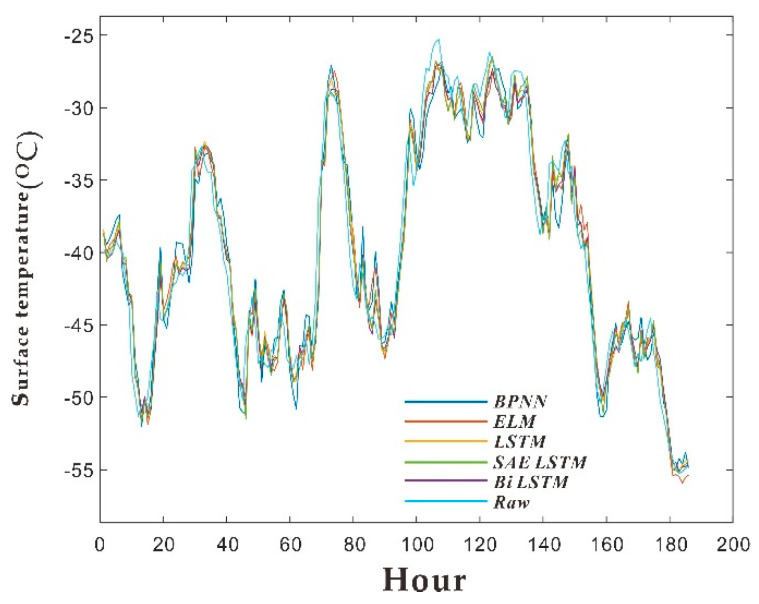
The test set performance of five prediction models for temperature prediction.

In order to verify the previous method and model, the most recent temperature observation data was selected. The data collection date is from February 24, 2020, to July 29, 2020, with a total of 953 temperature data (collect once every four hours). After training the prediction with short-term data, the five test set data are put together for comparison, as shown in Figure 14.

**Figure 15:**
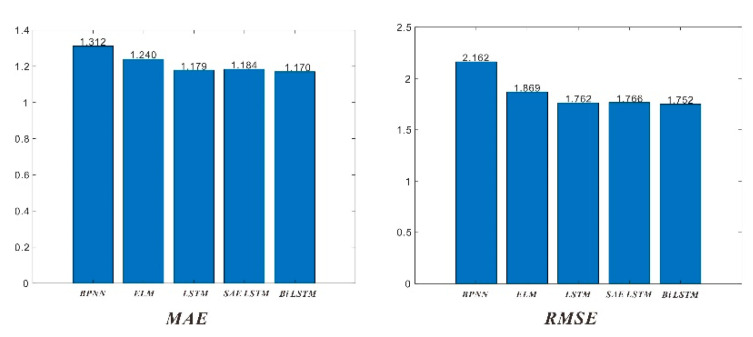
The test set performance of five prediction models for temperature prediction.

The evaluation parameters of each model including RMSE, MAE, U1, and U2 are shown in Figure 15:

It can be seen from the test set that due to the reduction in sample size, the relevant evaluation indicators have become worse than before. However, it is still in the same order of magnitude, and the two indicators of Bi LSTM are the lowest. Therefore, when using the prediction algorithm, the algorithm is still used to predict the temperature.

**Figure 16:**
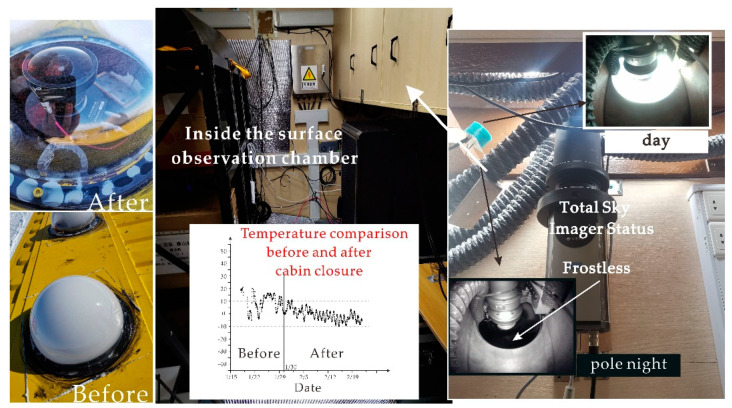
Cabin operation status comparison before and after adopting the strategy.

The most important indicators for evaluating the state of the system are cabin temperature, sensor collection rate, and data storage capacity. By monitoring the status of the ground observation cabin and the temperature data obtained in the cabin every day, it is most appropriate to get the sensor host at an ambient temperature of −10 to 0 °C, which can ensure the operation of the all-sky imaging sensor in the polar night. There is a small amount of frost on the dome cover of the all-sky imager, which does not affect the auroral observation. As shown in Figure 16.

The sealing time for the surface observation module is January 31, 2020. No one has entered since then, and the subsequent operating state is unattended. It can be seen that the temperature maintained by the internal ambient temperature sensor during the unattended process is between 0 and −10 °C. Due to the personnel activities during the initial installation of the main engine, a high-power 3000 W electric heater was installed in the cabin, so the temperature reached 20 °C. After entering the polar night, the internal monitoring images we observed after entering the polar night in April 2020 are shown in Figure 16. It can be seen that the observation cabin cover has less frost and does not affect the auroral observation. Moreover, the file storage and file information form show that all the sensors are working correctly.

The on-site implementation plan follows three principles:

1. The design needs to ensure the quality of the observation data of the complex sensor network.
2. Under the premise of guaranteeing observation, the energy system and control system need to operate stably.
3. It is necessary to ensure that the surrounding environment is not polluted and damaged.

**Figure 17:**
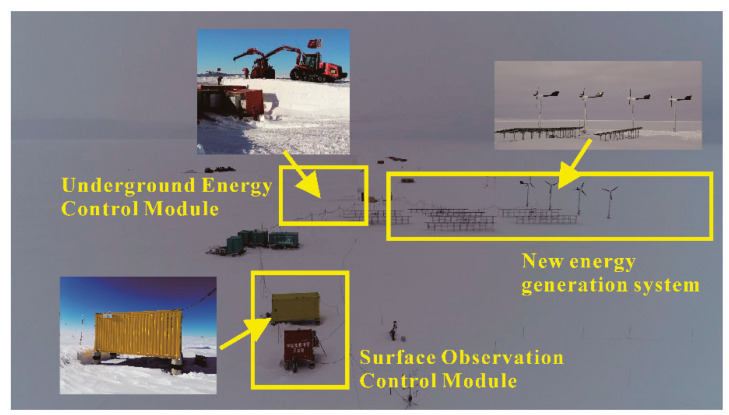
Aerial imagery of the entire Remote Control, Image Acquisition, Operation Maintenance, and Document Management (RIOD) system.

According to these three basic principles, the site layout of the main building was carried out on December 20, 2019. The entire system is divided into the surface control communication observation part, the underground energy part, and the surface new energy power generation part. Because the underground part has been landfilled, it is impossible to see the whole from the perspective of aerial photography. Figure 17 shows the aerial photography of the entire camp before the evacuation of personnel. In the figure, the underground energy control section is marked. The small picture shows the photos taken while laying the underground energy section. The position of the photovoltaic panel in the image and the direction of the surface cabin are placed according to the minimum contact surface of the annual average wind direction. This way can reduce heat loss, and the elevated box can prevent the accumulation of snow.

## 5. Conclusions

The purpose of this research is to find a relationship between on-site heating power and outdoor temperature, through neural network model training to obtain the optimal model for temperature prediction, combined with a heating strategy to ensure the stable operation of the on-site equipment. The following conclusions are drawn through the above results and experimental process:

(1) In the algorithm training experiment, based on different error indicators, the prediction accuracy of Bi LSTM is higher than that of the other four neural networks, especially in the annual data. This shows that Bi LSTM improves the shortcomings of LSTM for time series prediction.

(2) Field heating experiments have determined a set of experimental data of internal heating power corresponding to the air temperature when the outdoor wind speed is 10m/s. Equation (12) shows the relationship between input energy and temperature difference derived from the heat transfer model. The experimental data is combined to get Table 3, and combined with the optional internal heating method, the relationship between 8 groups of air temperature and power is determined. Besides, the discontinuous adjustment of heating power and the delay of remote-control satellite communications determine the necessity of predicting the temperature.

Accurate predictions of environmental parameters are of considerable significance to many activities on the Antarctic site. Climate predictions in small Antarctic areas can provide strong support for local scientific research activities. The establishment of a heat conduction network in traditional modelling is a standard method to control the target temperature threshold. This paper proposes an environmental maintenance method for a space physics observation module based on heat transfer physics model combined with a neural network prediction algorithm. In this paper, some advanced prediction models (such as Bi LSTM, SAE LSTM, etc.) are introduced into the experimental process, and the best prediction model is obtained by establishing evaluation indicators (such as RMSE). Combined with the traditional heat conduction network model, the energy of the system to maintain the system temperature can be determined, maintaining a constant temperature difference between indoor and outdoor per unit time. Combining these two models, a method for generating an on-site heating strategy operation ticket is proposed. This method and related hardware equipment can guarantee the operation of precision equipment (such as all-weather imagers) for astronomical observations and space physical observations in the Antarctic region. It provides an effective solution for other unmanned observation systems in the Antarctic area. The method is not limited to temperature prediction and heating. When combined with other physical models, the corresponding reference can also be provided based on the relevant observation data. The introduction of artificial intelligence algorithms can offer more solutions in this field. In future research, if more sample parameters (such as wind speed, and air pressure) are introduced to form a multiple-input single-output neural network and corresponding data processing (such as wavelet transform) is added, more accurate prediction and evaluation may be obtained.

## Figures and Tables

**Table 1:** Sensor information of the automatic weather station.

Equipment	Mode	Temperature Range	Communication Mode
Ultra-Sensitive Imaging	i Kon-M 934 Series, Oxford Instruments, Abingdon OX13 5QX, UK	−20~+25 °C	USB 2.0
Wide-Angle Monochromatic Imager	all-sky monochromatic imaging 557.7nm, Keo Scientific Calgary, Alberta, CAN	−80~+20 °C	USB 2.0
Wide-Angle Monochromatic Imager	all-sky monochromatic imaging 630.0nm, Keo Scientific Calgary, Alberta, CAN	−80~+20 °C	USB 2.0
GNSS ionospheric scintillator	CJW-1H, CETC Xin Xiang, HN, China	−40~+85 °C	USB 2.0
Flux-Gate Magnetometer	LEMI-025,LC ISR, Naukova St., Lviv, 79060, UKR	−5~+30 °C	RS-232
Induction coil magnetometer	LEMI-120,LC ISR, Naukova St., Lviv, 79060, UKR	−10~50 °C	RS-232
NAS	TS869L, QNAP, Xintai 5th Rd, Xizhi City, Taipei, China	0~50 °C	USB 2.0& USB 3.0&HDMI
Network switches	TL-SG3226, TP-LINK, Guang Ming New District, Shenzhen, GD China	−40~70 °C	Base-T RJ45
GPS timing devices	DNS-8, Neutron, Tong Zhou District, BJ, China	−40~85 °C	Base-T RJ45

**Table 2:** Sensor information of the automatic weather station.

Sensors	Mode	Temperature Range	Communication Mode	Measurement Accuracy
Wind speed	05103-45 R.M.Young, Traverse City, MI, USA	−40~60 °C	RS-232	0.5 m/s
Wind direction	05103-45, R.M.Young, Traverse City, MI, USA	−60~30 °C	RS-485	0.3°
Temperature sensor	DS28EA00, DALLAS Sunnyvale, CA, USA	−40~+85 °C	Single bus	0.5 °C
Ranging sonar	SR50AL, Campbell Scientific Logan City, Utah, USA	−45~+50 °C	RS-232	0.5 cm
Pyranometer	FR-TBQ, Purple Forain Wuhan, Hu Bei, CHN	−20~+40 °C	RS-232	9.167μ v/W·m-2

**Table 3:** Corresponding heating power table under different temperature differences.

Temperature Difference (°C)	Input to the System Over 1 h Heat (KJ)	Heating Power (W)
0–5	0–344.7	0–95.75
5–10	344.7–689.4	95.75–191.5
10–15	689.4–1034.1	191.5–287.25
15–20	1034.1–1378.8	287.25–383
20–25	1378.8–1723.5	383–478.75
25–30	1723.5–2068.2	478.75–574.5
30–35	2068.2–2412.9	574.5–670.25
35–40	2412.9–2757.6	670.25–766

**Table 4:** Properties of materials in heat conduction networks.

Materials	$\mathrm{Specific}\;\mathrm{Heat}\;\mathrm{Capacity}\;c\left(kJ/\left(kg\times {\;}^{{}^{\circ}}C\right)\right)$	Density $\rho \left(kg/m^{3}\right)$	Heat Transfer Coefficient λ(W/m ⋅ °C)
Air	1.4036	1.4527	0.02234
Cyanuramide,2,4,6-Triamino-s-Triazine	0.9707	1095.27	0.159
Asbestine	0.84	[140,200]	0.043
PU	0.75	125	0.024

**Table 5:** Computer configuration.

Name	Settings
Hardware	Desktop computer
CPU	Intel(R) Core (TM) I5-9600K 3.70GHz
RAM	DDR4 8G 2666MHz
Hard drive	2T
Software& Operating system	MATLAB R2019a, Windows 10

**Table 6:** Parameter setting of the neural networks.

Algorithm	Parameter Settings
BP	ME = 1000, GT = 1.0 × 10**^−7^**, HL1 = 20, HL2 = 20. AF = S
ELM	ME = 1000, AF = S
LSTM	ME = 250, GT = 1, ILR = 0.01, LRDP = 125, LRDF = 0.1, V = 0, L2R = 0.0001
Bi LSTM	ME = 250, GT = 1, ILR = 0.01, LRDP = 125, LRDF = 0.1, V = 0, L2R = 0.0001
SAE-LSTM	ME = 250, GT = 1, ILR = 0.01, LRDP = 125, LRDF = 0.1, V = 0, ETF = satlin, DTF = purelin, L2R = 0.0001, L2WR = 4, SP = 0.6

## Acronyms

RIOD	Remote Control, Image Acquisition, Operation Maintenance and Document Management System
PDU	Power Distribution Unit
PB300	Pisten Bully–300 Polar
PRIC	Polar Research Institute of China
TYUT	Taiyuan University of Technology
NAS	Network Attached Storage
BP	Back Propagation
ELM	Extreme Learning Machine
LSTM	Long Short-Term Memory
SAE LSTM	Stacked Auto Encoder Long Short-Term Memory
Bi LSTM	Bi-directional Long Short-Term Memory
